# Coherent Superposition in Grating-Based Directional Dark-Field Imaging

**DOI:** 10.1371/journal.pone.0061268

**Published:** 2013-04-23

**Authors:** Andreas Malecki, Guillaume Potdevin, Thomas Biernath, Elena Eggl, Eduardo Grande Garcia, Thomas Baum, Peter B. Noël, Jan S. Bauer, Franz Pfeiffer

**Affiliations:** 1 Department of Physics and Institute of Medical Engineering, Technische Universität München, Garching, Bavaria, Germany; 2 Institut für Radiologie, Klinikum rechts der Isar, Technische Universität München, München, Bavaria, Germany; UC Davis School of Medicine, United States of America

## Abstract

X-ray dark-field scatter imaging allows to gain information on the average local direction and anisotropy of micro-structural features in a sample well below the actual detector resolution. For thin samples the morphological interpretation of the signal is straight forward, provided that only one average orientation of sub-pixel features is present in the specimen. For thick samples, however, where the x-ray beam may pass structures of many different orientations and dimensions, this simple assumption in general does not hold and a quantitative description of the resulting directional dark-field signal is required to draw deductions on the morphology. Here we present a description of the signal formation for thick samples with many overlying structures and show its validity in experiment. In contrast to existing experimental work this description follows from theoretical predictions of a numerical study using a Fourier optics approach. One can easily extend this description and perform a quantitative structural analysis of clinical or materials science samples with directional dark-field imaging or even direction-dependent dark-field CT.

## Introduction

X-ray grating interferometry is a very promising candidate to improve current imaging techniques in materials science and medicine without the large effort of creating completely new machines [1]–[4]. Besides transmission and phase-contrast imaging it provides a new contrast channel, which is well-known from visible light and electron microscopy: The dark-field contrast [5]. Without requiring high resolution detectors that are not practicable in the medical field because of higher dose requirements, dark-field radiography and tomography allow to draw conclusions about morphological parameters of sub-pixel size structures such as their dimensions, location, and orientation [6]–[15]. One method to retrieve information about the sub-pixel structure orientation and anisotropy is directional dark-field imaging [16], [17].

The dark-field contrast signal 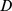 is extracted from the visibility of the interference pattern of the grating interferometer. The more the interference pattern is distorted and its visibility decreases the stronger is the measured dark-field signal. As the interferometer is built from gratings made of one-dimensional lines and spaces, it is only sensitive to distortions that occur perpendicular to these lines. This direction-dependence is utilized in directional dark-field imaging. Here the dark-field signal is examined for different orientations of the sample relative to the interferometer lines. By rotating the sample and recording the dark-field signal 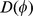 with respect to its orientation 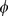 it becomes possible to draw conclusions on the anisotropy of the sub-pixel size structures as it will be described further below.

For thin specimens the x-ray beam passes only through a few structures located behind each other. Because of that, almost all structures that contribute to the dark-field signal in each single detector pixel can be assumed to be parallel and it is easy to deduce their direction and anisotropy from the orientations producing the maximum signal. Up to now it is unknown how the recorded directional dark-field signal is formed for thick samples. Here several differently oriented layers of substructures can lie behind each other, each producing its own direction-dependent signal. Knowing the physics behind the signal formation is a fundamental prerequisite for potential future medical diagnostic and materials science applications as well as direction-dependent computed tomography methods.

In this study we show how the x-ray directional dark-field signal of the superposition of arbitrarily oriented structure layers is related to the signal created by each component. In contrast to existing experimental work [18] our study is theoretically based on a Fourier optics approach realized in numerical simulations. From these predictions we derive a theoretical model describing the superposition principle, which is related to the setup parameters. We cross-check the validity of this model qualitatively and quantitatively with experimental data of samples containing a large number of highly oriented sub-pixel size structures.

## Results and Discussion

**Figure 1:**
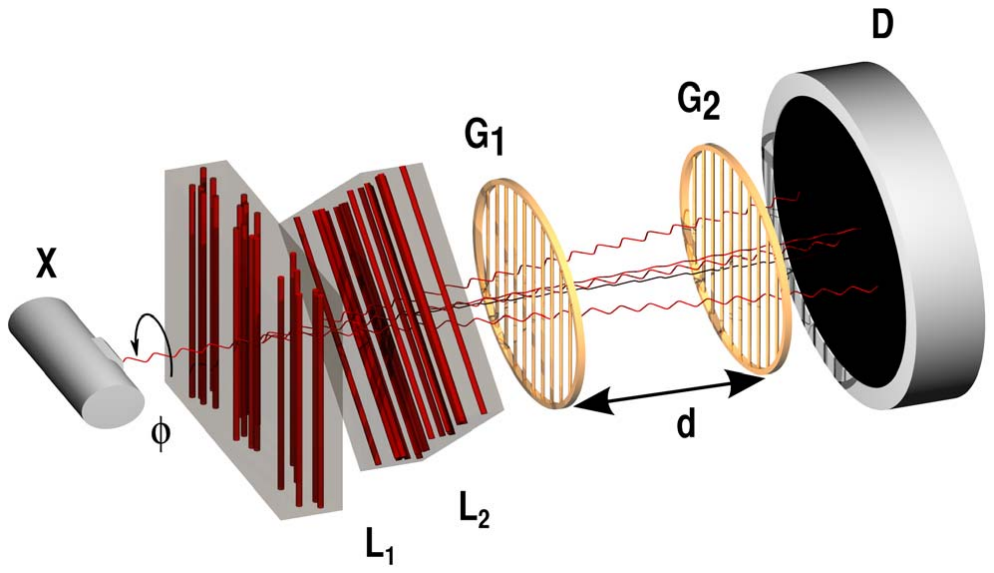
Schematics of the x-ray grating interferometry setup used to study the superposition principle in directional dark-field imaging. The simulated setup consisted of an x-ray source (X) two layers of highly oriented structures (cylinders, 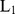 and 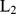), the grating interferometer (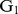 and 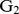) and a detector (D). For the experiment an additional grating (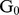) was used right behind the source to enforce the coherence conditions required by the interferometer. The sample layers were rotated independently from each other around the optical axis to retrieve the direction-dependence of the dark-field signal.

Let us first focus on the theoretical predictions for the superposition of directional dark-field signals drawn from simulations (cf. [15]). For that purpose we simulated a complete but simplified x-ray grating interferometry setup for directional dark-field imaging (see fig. 1). It consisted of a monochromatic x-ray source (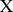) emitting plane waves, two sample layers (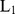 and 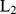), the grating interferometer (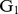 and 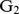) and a detector 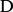. Here we considered a sample consisting of 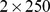 parallel cylinders all lying in two separate planes perpendicular to the beam axis and one behind the other. Each cylinder then has a well-defined scattering direction perpendicular to the cylinder axis. In consequence we expected the signal to be completely anisotropic meaning a strong cosine oscillation with respect to the angle of rotation around the beam axis as it was found previously in experiments [16], [17]. The cylinder positions were randomly distributed over an area of 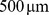 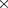 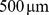, which was covered by 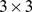 detector pixels. At first each layer was simulated separately to retrieve its independent contribution to the dark-field signal. To get the directional information we rotated the sample in discrete steps of 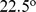 around the beam axis. The angle of rotation 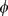 is measured between the horizontal axis and the cylinder axis. Afterwards both sample layers were simulated together for certain constant relative angles 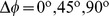 in between the two of them. In addition to that, the 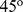 orientation was simulated with the first layer containing only 125 cylinders corresponding to half the thickness.

**Figure 2:**
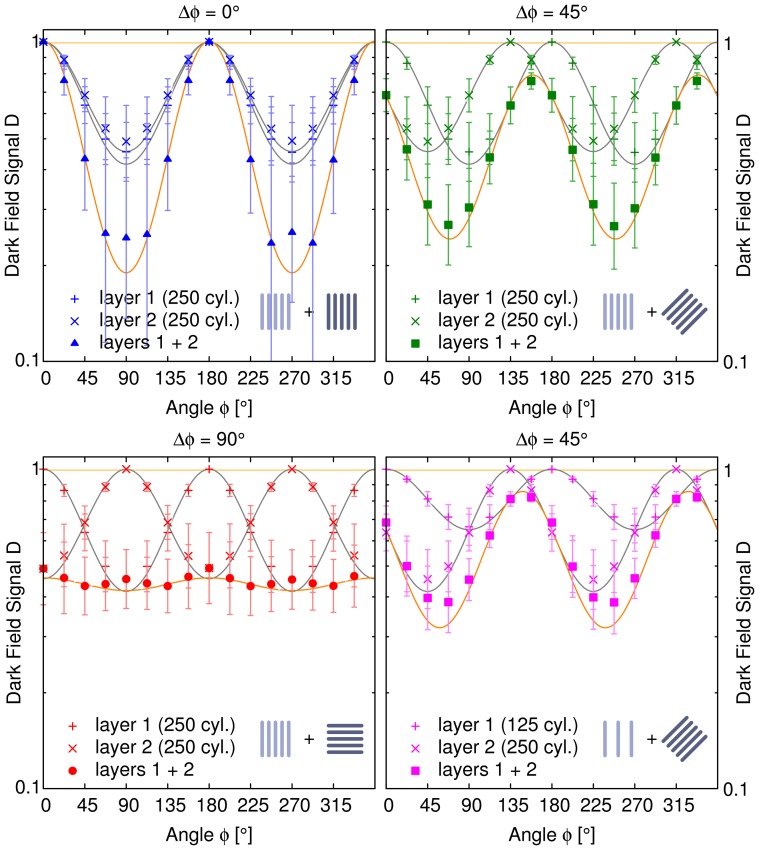
Directional dark-field simulation results for three different relative orientations of two strongly oriented sample layers. The upper row and left column both show the dark-field signal with respect to the orientation of two sample layers containing 250 cylinders each. The cylinders were randomly distributed over a plane perpendicular to the x-ray beam. We examined different relative orientations between the layers 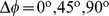. For each relative orientation the plots show the dark-field signals of the separate layers and both of them together. Clearly visible is the 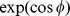 dependence for each single layer and the superposition of both of them. For the single layer results we calculated the corresponding fit curves shown as solid lines. The product of both fit functions is shown as well as a solid line. It perfectly agrees with the simulation results for both layers together, although in the upper left plot the variance of the sum signal with respect to the actual microstate of the cylinder ensemble is quite large. In the lower right plot the first sample layer contained only 125 cylinders to simulate a layer of half the thickness. The shape of the signals does not change. From these results we derived that the superposition signal is simply the product (logarithmic scale) of the two single layers. As a simple consequence for a relative angle of 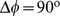 the oscillation almost completely vanishes and the whole sample appears nearly perfectly isotropic.

Each single sample layer on its own produces a signal, which follows an 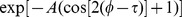 dependence (see fig. 2). This is visualized by the corresponding fit curves shown in addition to the data points. The constant parameter 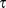 gives the orientation causing the strongest signal (lowest values). 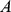 is related to the amount of direction-dependent scattering and depends on the setup parameters as well as the structure dimensions inside the sample. The maximum signal is reached for orientations where the cylinders are oriented parallel to the direction of the interferometer's grating lines. Analogously, when the cylinders are orientated perpendicular to the grating lines the dark-field signal vanishes completely. From the cosine curves the average logarithmic dark-field signal and the anisotropy with respect to the angle of rotation can be calculated. The average signal is equal to the constant offset of the cosine curves. The anisotropy then is calculated from the amplitude of the oscillation relative to this offset. Consequently for a completely anisotropic signal the amplitude of the cosine is equal to its offset.

The signal of both layers together shows the same angular dependence as the two single layers but with varying amplitude and phase depending on the relative orientation and the amplitudes of the signal of each single layer. When the orientations of the structures in both layers are chosen to be parallel, the sum signal only changes in amplitude with respect to the individual signals. For a relative angle of 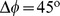 between the two layers the superposition signal has its maximum right in the middle between the maxima produced by the separate layers and it has a slightly larger amplitude. When the structures inside the two layers are oriented perpendicular to each other the resulting directional dark-field signal is almost constant. The remaining oscillations originate in the randomly distributed locations of the cylinders. Finally if the first layer contains less scatterers the phase of the sum signal is less affected and consequently approaches the phase of the dominating signal produced by layer 2. For the case of both sample layers oriented in parallel the variance of the superposition signal with respect to the actual microstate of the statistical ensemble of cylinders is quite large. We ascribe this to the high correlation between both layers and the grating lines.

### Model for the superposition principle

From these findings we derived a physical model predicting the superposition principle of x-ray directional dark-field imaging. The directional dark-field signal of each single layer 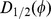 is given by
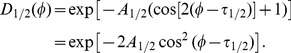
(1)


Here 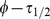 is equal to the angle between the direction of scattering of the sample and the direction of maximum sensitivity of the interferometer.

Starting from this we can express the scattering caused by each sample layer with a two-dimensional vector 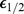 with its magnitude corresponding to the scattering strength and its orientation giving the direction of scattering. The interferometer itself is only sensitive to scattering perpendicular to the orientation of the grating lines. This as well can be expressed by a two-dimensional vector quantity 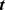 with its magnitude being the sensitivity of the interferometer in the direction of 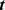. With these prerequisites we reformulate eq. (1) and express the dark-field signal by
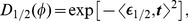
(2)with 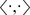 denoting the two-dimensional scalar product and 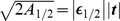.

From the plots in fig. 2 and the observations described above we infer that the superposition signal is given by the product of both single layers:
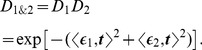
(3)


This equation describes the directional dark-field superposition principle for two completely anisotropic sample layers passed by an x-ray beam one after each other. An arbitrary number of further layers can be added by applying eq. (3) several times, which yields the product of the individual direction-dependent dark-field signals of each layer.

One immediate consequence of this equation is that the superposition of two orthogonal structures of equal scattering strength results in a constant scattering profile with respect to the rotation angle. In directional dark-field imaging such a sample will appear isotropic. Furthermore every directional dark-field signal will be a superposition of harmonics of the same angular period and consequently be another harmonic with identical period. This kind of superposition in contrast to small-angle x-ray scattering (SAXS) can be regarded coherent.

For two arbitrary scattering directions and strengths the logarithmic sum signal can be calculated by simply adding two harmonics. We predicted the results for the superposition signal by multiplying the fit curves of the single sample layers, which is equivalent to that. The expected curves are shown in fig. 2 as well and the data points agree with this theoretical model.

Generalizing eq. (3) to a sample consisting of infinitesimally thin scattering layers leads to
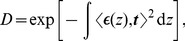
(4)the line integral for anisotropic scattering samples. In this model an isotropic scatterer can be represented by the superposition of two perpendicular scattering directions causing a dark-field signal that is independent of the sample orientation. For a mixed isotropic and anisotropic sample this leads to an additional constant offset of the cosine curves in the simulated/measured data.

### Experimental realization by a well-defined sample

To realize the simulated situation in experiment we chose a specimen composed of two wooden skewers located one behind the other in the x-ray beam. For these samples scattering mainly occurs in directions perpendicular to the skewer axis because of the numerous wooden fibers oriented along this direction. For the experimental demonstration, we utilized a more involved setup than the simplified one used for the simulations above. We used a tube source for illumination, so that we needed to introduce a source grating (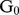) to meet the coherence requirements of the setup. Because of this the illumination was polychromatic as well and the x-ray beam was divergent. Nevertheless with respect to the physical effects in the sample and the setup these changes did not affect the experimental results significantly. The angular area occupied by the sample was small enough to neglect the beam divergence. The scattering strength in the sample differs for the various photon energies, but concerning the measured orientation and anisotropy every part of the spectrum contributes in the same way.

**Figure 3:**
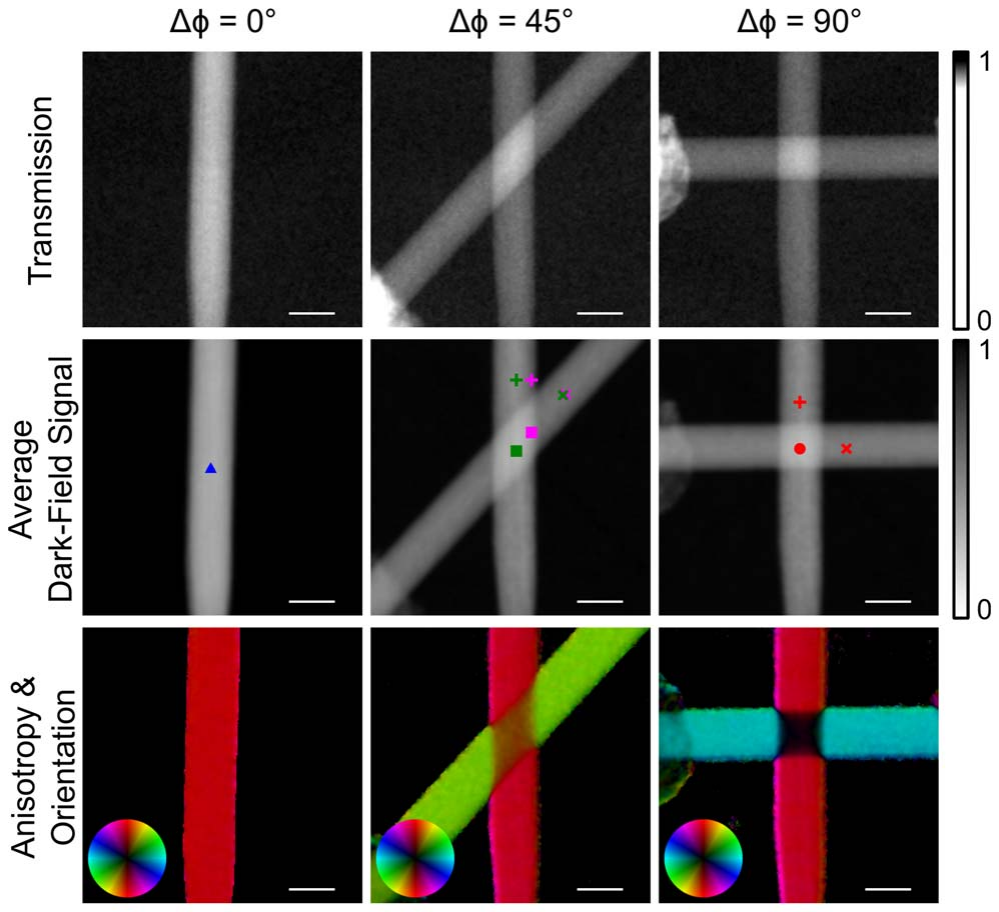
Experimental realization of the simulated scenario of differently oriented layers on top of each other. Two wooden skewers were put on top of each other and examined with respect to different relative orientations. Because the fibers of the wood form highly oriented substructures parallel to one skewer's central axis each skewer strongly scatters in a well-defined direction perpendicular to its central axis. The color scale has been adapted to increase the contrast for the transmission images. Compared to the rather weak absorption of the material the dark-field signal clearly predominates for this kind of sample. Shown in the middle row, the mean value of the dark-field signal with respect to the rotation simply is the product of the mean values of each skewer, as it is for the transmission depicted above. In the bottom row the anisotropy images encode the direction of the structures in the color and the degree of anisotropy in the brightness. For completely parallel orientation of the two skewers (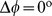) the superposition shows the same orientation. For an angle of 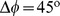 between them, the resulting signal depends on the amount of material penetrated by the beam. Wherever both thicknesses are equal the measured orientation is exactly the circular mean of both individual orientations. Otherwise intermediate orientations appear wherever the penetrated thickness of one of both skewers is larger than for the other. For an angle of 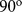 and equal thicknesses the anisotropy signal completely cancels out. Length of the scalebar: 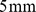.

Fig. 3 displays the transmission, average dark-field and anisotropy signals for three different relative orientations (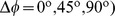 of the two skewers. Here the structure orientation is encoded in color, while the anisotropy is given by the brightness. Qualitatively the transmission as well as the average dark-field signal simply multiply to form the superposed signal. As expected from the derived model, for comparable sample thickness the orientation is equal to the mean direction. This corresponds to a roughly equal amount of scattering structures in both skewers at this location. For perpendicular scattering directions the anisotropy is strongly suppressed.

### Quantitative check of the model

**Figure 4:**
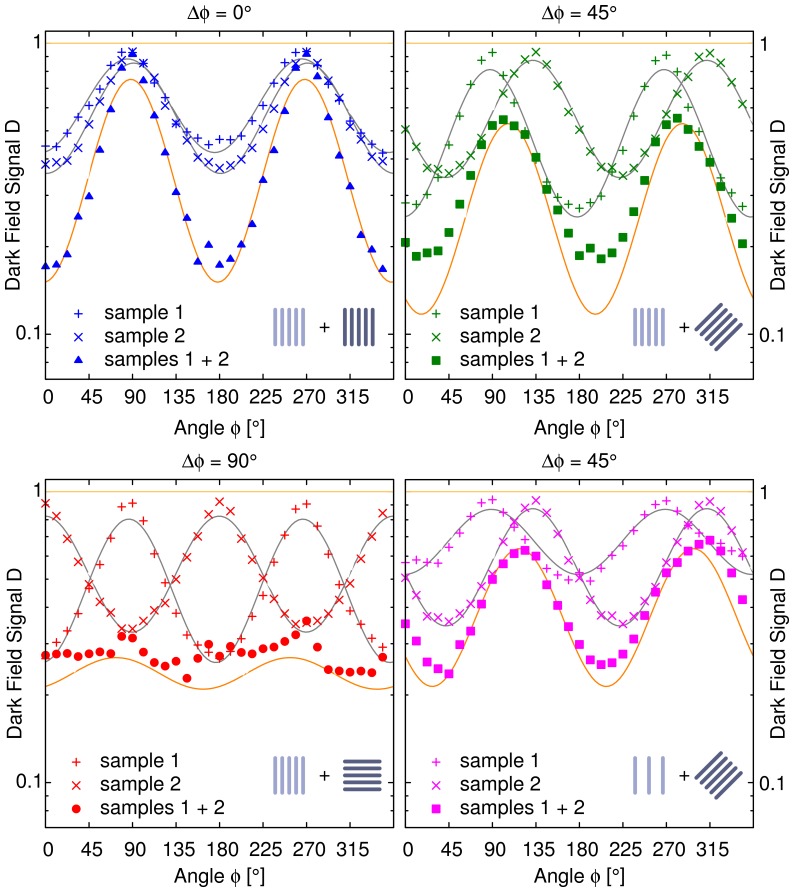
Quantitative evaluation of the superposition principle at specific locations of the combined samples. The positions from which the curves were taken correspond to locations on the wooden skewers, where either one of them were penetrated by the x-ray beam or both after each other. They are marked in fig. 3 by the corresponding symbols. For the parallel oriented samples the single layer data were taken from separate scans of the single samples. To test the validity of the model derived from the simulation results fit curves were calculated for the single samples shown in gray. The predicted superposition signals resulting from the fit parameters and the model are shown in orange color. The direction-dependent curves for the superposition of both samples coincide with the predictions drawn from the model and both single curves for every relative orientation between the two wooden skewers.

A quantitative examination of several representative locations taken from the example in fig. 3 is shown in fig. 4. Each single directional dark-field signal obeys eq. (4). Due to a remaining isotropic contribution caused by wood fibers that are not oriented along the skewer axis the curves show a small additional offset. We derived the model parameters from these two curves by fitting the model to the data (shown in gray). From the directional dark-field signal of each single sample and the superposition principle derived above we calculated the expected values for the superposition of both sample layers (shown in orange). The measured superposition signal follows the model in all cases, although some deviations remain. They originate in slight variations between the different locations from which the curves are taken.

For a relative orientation of 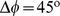 the curves on the one hand show the results for a location where both sample layers have almost identical thickness and on the other hand for a point moved a bit sideways such that the first layer has almost half the thickness. Here the locations of the superposition curve's extrema clearly move towards the locations of those of the skewer, which has the stronger scattering contribution. As the scattering mainly depends on the amount of scatterers this is the skewer, which is thicker at the chosen location.

### Application to clinically relevant specimens

**Figure 5:**
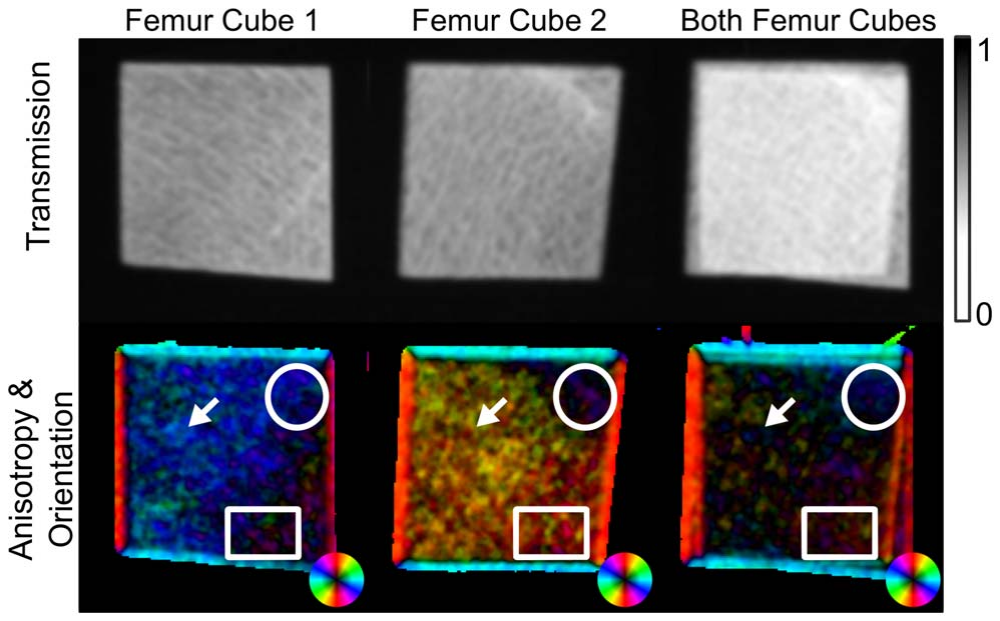
A clinically relevant example for the application of the derived model. Here the two sample layers consist of human femoral bone cut in cubes. Both cubes were each measured separately with the beam penetrating cube 1 in the anterior-posterior and cube 2 in the medial-lateral direction, and then once again when both cubes were placed on top of each other but oriented in the same way. The upper image row shows the resulting transmission images and the lower row the measured orientation and anisotropy. As before, the anisotropy images encode the direction of the structures in the color and the degree of anisotropy in the brightness. Depending on the local amount of anisotropy in both separate samples the local direction of the combined sample is either dominated by one of the components (regions marked with circle and rectangle) or the anisotropy is strongly reduced because the average structure orientations are perpendicular to each other between both cubes.

Fig. 5 shows a biomedical example for the superposition principle: Transmission and directional dark-field images of two human trabecular bone cubes, which were harvested from the femoral head of one female individual. In contrast to cortical bone, trabecular bone is a sponge-like structure of calcified bone matrix. The trabecular microstructure is aligned with the principle stress trajectories. Consequently there exist preferred structure orientations depending on the anatomical location inside the femur.

The two cubes were once measured separately with the viewing direction aligned along the anterior-posterior (cube 1) and the medial-lateral direction (cube 2). In the first case the superior-inferior direction was pointing upward, while in the second case it was the anterior-posterior direction. This was kept constant for the whole experiment.

Both femoral cubes showed a distinct preferred orientation as expected (cf. fig. 5, left and middle column). Their structures pointed roughly in perpendicular directions. In addition to that another measurement was carried out, where both cubes were put on top of each other (cf. fig. 5, right column).

Depending on the amount of anisotropy found in each separate cube the directional dark-field image of the combined sample shows a preferred direction that is more or less equal to the dominating component. Two such areas are marked by a circle (dominated by cube 1) and a rectangle (dominated by cube 2). A large fraction of the area that is occupied by both cubes in the combined sample shows less if no anisotropy at all. This follows directly from the superposition model we derived above and the perpendicular orientation of the structures between both cubes.

## Conclusion and Outlook

In summary we have shown how the directional dark-field signal drawn from x-ray grating interferometry can be calculated for samples that are composed of highly anisotropic layers of differing orientation. Starting from theoretical predictions gained from simulations we developed a model for the superposition of the signals originating in two different sample slices: The harmonic oscillations observed for the single layers can simply be added to retrieve the superposition signal. As a direct consequence, layers with perpendicular orientation and similar scattering strength will show no anisotropy in dark-field imaging. We checked the correctness of this model by means of experimental data and found very good agreement.

With this model at hand it is now possible to predict and describe the directional dark-field signal for thick samples containing more than one layer of oriented scatterers. This could for example be applied in materials science (e.g. compounds containing carbon fibers or meshes) and even have a large impact on medical diagnostics for example in the case of trabecular bone as we have pointed out in this study. For instance, diagnosis and treatment monitoring of osteoporosis, which is major public health problem through its association with fragility fractures, may be improved by taking interferometric projection images. Osteoporosis is characterized not only by loss of bone mineral density but also by decreasing bone quality including a deterioration of the trabecular microstructure [19]. Such changes of the trabecular bone structure due to osteoporosis or osteoporosis-related treatment may be detected a lot earlier and with higher sensitivity if compared to conventional x-ray techniques. Furthermore we will extend this model to perform direction-dependent computed tomography based on the dark-field signal. This will allow to reconstruct the sub-pixel structure orientation and anisotropy with respect to the exact anatomical location.

## Materials and Methods

The simulations described in this article were performed using a Fourier optics [20] approach and a simplified representation of the setup shown schematically in fig. 1. We used a similar approach to the one we described earlier in [15] taking into account the actual experimental setup. Starting point of the simulation was the x-ray source represented by a monochromatic plane wave with a photon energy of 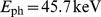. The wavefront was discretized at 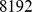 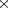 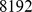 grid points that were 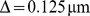 apart. From this point on angular spectrum propagation [21] was used to calculate the x-ray wavefront at certain positions behind the source. For every setup component we used its complex amplitude transmission function (projection approximation) to simulate the effects on the incoming wavefront. The sample contained two separate layers of long parallel cylinders at random positions distributed over an area of 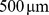 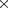 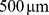 around the beam axis and oriented perpendicular to the beam direction. Each cylinder consisted of calcium (density 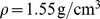) and had a diameter of 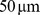 and a height of 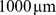. Each sample layer was applied to the wavefront separately with a propagation step in between. The two gratings 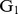 (phase grating) and 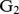 (analyzer grating) had a period of 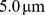 and a duty cycle of 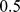 (Ronchi ruling). The height of the phase grating lines was 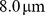, which is equivalent to a phase shift of 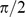 at this photon energy. The analyzer grating was located 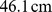 behind 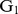, which corresponds to the first fractional Talbot distance at this energy. Its lines were made of 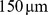 of gold to ensure high absorption and visibility. The grating lines were oriented along the vertical direction at all times. Right after the last grating followed a pixelated photon counting detector with a pixel size of 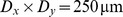 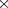 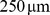. Consequently – because of the much greater height of the cylinders in the sample – we avoided border effects of the cylinder endcaps. The finite focal spot size was taken into account by smoothing the intensity of the wavefront right behind the analyzer grating with a Gaussian filter kernel of width 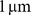. The two sample layers (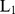 and 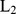) were located 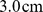 and 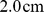 in front of the phase grating 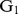. For each simulation run they were rotated independently for an angle 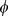 around the x-ray beam axis. For each sample orientation a stepping curve was recorded by moving the analyzer grating (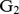) in horizontal direction over one grating period in 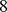 steps (phase stepping). The simulated area covered roughly 5×5 detector pixels, but only the center pixel provided the dark-field data used in this article. Each simulated orientation was repeated 9 times with different random positions of the cylinders. This allowed to estimate the average and spread of the dark-field values. We calculated the components of the complex refractive index needed for the setup components with an adapted version of *mucal* written by Bandyopadhyay and Segre [22] (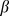) and as described in [23] (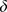).

The experiments were performed at a laboratory setup at the Technische Universität München consisting of a High Power X-Ray tube (MXR-160HP/11 by COMET AG, Switzerland) at an acceleration voltage of 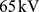 and a current of 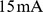 with a 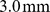 aluminium filter. The gratings consisted of two absorption gratings (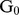 and 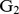) with a silicon substrate height of 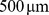 and 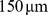 and 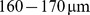 high gold lines filled with SU-8 in between. The phase grating was made of 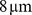 nickel lines on a 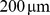 thick silicon substrate. The period of the gratings was 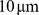 for the absorption gratings and 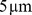 for the phase grating and their duty cycle was 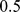. A Varian PaxScan 2520D with a CsI scintillator served as x-ray detector.

The wooden skewer and femur cube samples were measured in air while mounted on a plexiglass (PMMA) panel, which was fixed inside an Eulerian cradle manufactured by Huber Diffraktionstechnik GmbH & Co. KG, Germany. This goniometer allowed rotation of the samples around the beam axis. The distances between the gratings were 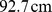, which corresponds to a design energy of 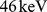 of the interferometer. The samples were located 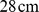 downstream from the phase grating and we ensured that their dark-field signal never reached saturation.

Both cubical bone specimens (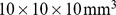) were harvested from the head of a fresh frozen human femur (62 year-old female, 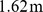, 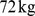). The femur was scanned with a 256 row multidetector CT (Philips Medical Care, Best, Netherlands) and no pathological lesions were observed on the macroscopic scale. Previous to the cutting process, the center of the femoral head and neck were mechanically defined. The specimens were cut from the center of the femoral head according to the anatomical direction of the femoral neck (cf. fig. S1). They were irrigated with 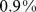 saline during machining using a low speed diamond saw (Dia Tech, Dia BS200, GmbH, Stuttgart, Germany). Thereafter, the bone specimens were defatted chemically by submerging them in ethanol and acetone to allow storage for all imaging procedures. The donor had dedicated her body for educational and research purposes to the local Institute of Anatomy prior to death, in compliance with local institutional and legislative requirements. Written informed consent was obtained from the donor. The study was reviewed and approved by the local institutional review boards (Ethikkommission der Fakultät für Medizin der Technischen Universität München, Germany).

We calculated the three different contrast signals from the Fourier transform of the resulting intensity variation as it was described earlier in the literature [2], [5], [15]. For the calculations of the directional dark-field signal we deviated from earlier descriptions [16] by using the logarithmic dark-field signal for the calculation of the average dark-field signal and the main orientiation. We applied a threshold when calculating the anisotropy data, such that only data points with an average dark-field signal stronger than 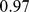 (wooden skewers) or 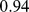 (femur cubes) were taken into account.

## Supporting Information

Figure S1 **Illustration for the harvesting process of the femoral bone cubes.** The original anatomical locations and orientations of both cubes are marked with their indices.
(TIF)
