# Correction: Coherent Superposition in Grating-Based Directional Dark-Field Imaging

**DOI:** 10.1371/annotation/66e71755-e00d-414c-b76e-1d7860c29f90

**Published:** 2013-11-18

**Authors:** Andreas Malecki, Guillaume Potdevin, Thomas Biernath, Elena Eggl, Eduardo Grande Garcia, Thomas Baum, Peter B. Noël, Jan S. Bauer, Franz Pfeiffer

Errors were introduced during the preparation of this article for publication. This article has been republished to replace corrupt figures. 

