# Supplementary figures and images for: Coherent Superposition in Grating-Based Directional Dark-Field Imaging
Source: PLoS One. 2013 Apr 23;8(4):e61268. doi: 10.1371/journal.pone.0061268 (PMC3634061; doi:10.1371/journal.pone.0061268)

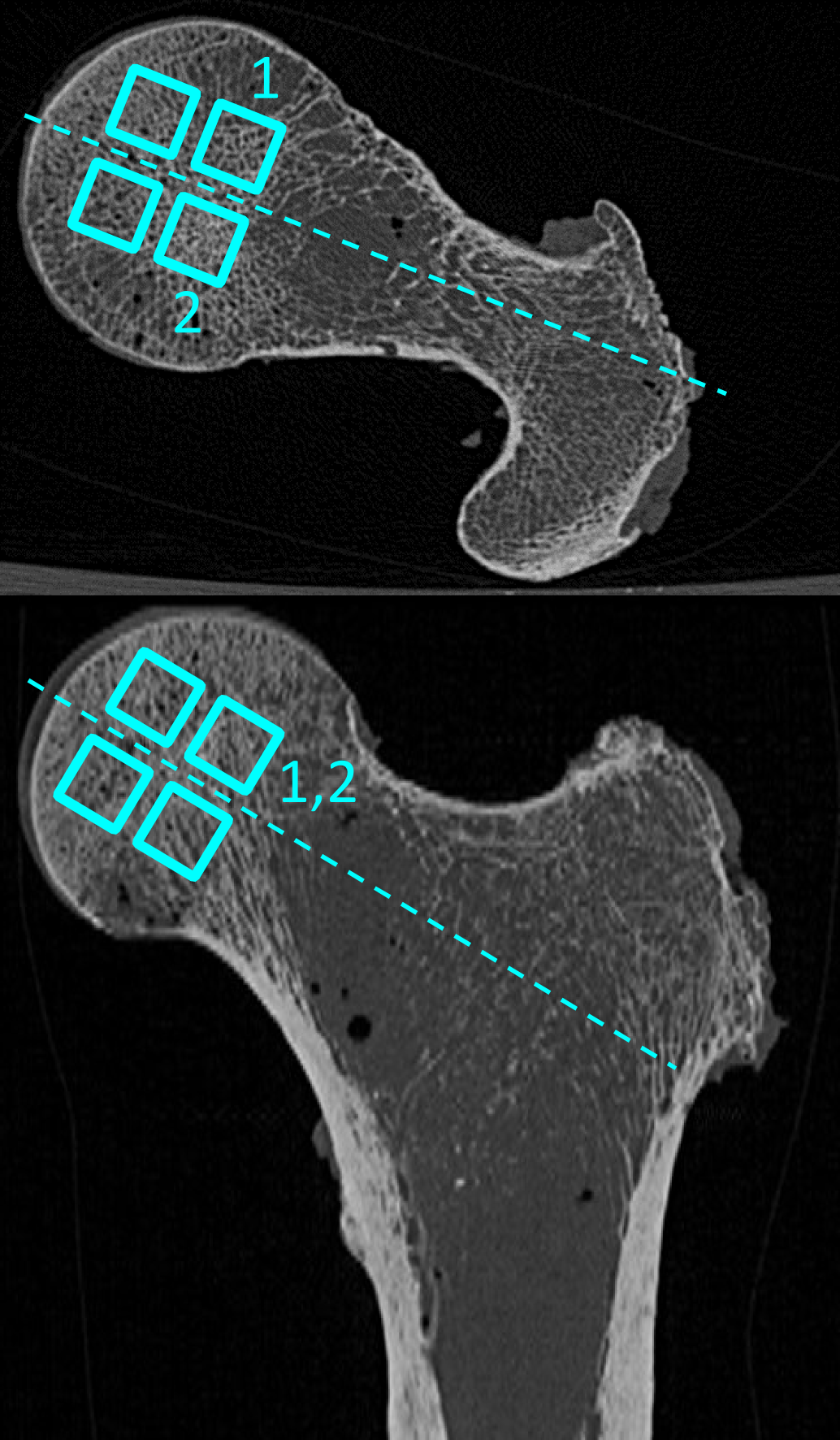

Supplement: Figure S1 — Illustration for the harvesting process of the femoral bone cubes. The original anatomical locations and orientations of both cubes are marked with their indices. (TIF) [file pone.0061268.s001.tif]
